# Efficacy and safety of traditional Chinese medicine combined with azithromycin sequential therapy for *mycoplasma* pneumonia among children: a meta-analysis of randomized controlled trials

**DOI:** 10.3389/fphar.2024.1431706

**Published:** 2024-10-17

**Authors:** Jing Lyu, Fei Fan, Ji Li, Qiong Wang, Xue Tian, Jiaxing Xu, Si Zhang, Bo Wang

**Affiliations:** Department of Paediatrics, Guang’anmen Hospital, China Academy of Chinese Medical Sciences, Beijing, China

**Keywords:** *Mycoplasma* pneumonia, children, traditional Chinese medicine formula, azithromycin, meta-analysis, randomized controlled trial

## Abstract

**Background:**

Traditional Chinese medicine (TCM) is used to treat *mycoplasma* pneumonia (MP) in children with favorable treatment outcome in China. In the present study, we evaluated the clinical efficacy of TCM combined with azithromycin (AZM) for the treatment of MP among children, providing high evidence-based reference for clinical treatment.

**Method:**

We retrieved eligible randomized controlled trials (RCTs) from CQVIP, CNKI, WanFang, NSTL, PubMed, Embase, and Embase databases from January 2000 to November 2023. Data extraction and quality assessment of the enrolled studies were independently by two reviewers. Review Manager 5.3 was used for meta-analysis.

**Result:**

A total of 51 RCTs involving 5,799 children aged 1–14 enrolled. Meta-analysis demonstrated that TCM combined with AZM improved the cure rate (odds ratio [OR] = 2.34, 95% CI: 2.06 to 2.64) and the effective rate (OR = 5.21, 95% CI: 4.22 to 6.43), shorted the disappearance duration of cough (WMD = −1.62, 95% CI: −1.90 to −1.34), the duration of fever (WMD = −1.62, 95% CI: −1.96 to −1.29), and the disappearance time of lung rales (WMD = −1.15, 95% CI: −1.32 to −0.98), improved CRP levels (WMD = −2.06, 95% CI: −2.57 to −1.55), IL-6 levels (WMD = −1.92,95% CI: −2.51 to −1.34), and TNF-α levels (WMD = −1.59, 95% CI: −2.14 to −1.04), and reduced adverse reactions (OR = 0.37, 95% CI: 0.32 to 0.44).

**Conclusion:**

TCM combined with AZM in the treatment of MP among children has favorable clinical efficacy and safety.

## Introduction

*Mycoplasma* pneumonia (MP) is common in children (Li et al., 2023), and its incidence rate has increased significantly in recent years, showing pandemic-level trends.

In traditional Chinese medicine (TCM), the occurrence of MP is associated with wind cold invading the lungs, phlegm heat obstructing the lungs, yin deficiency and lung dryness, and phlegm turbidity accumulation (Poddighe et al., 2022; Tsai et al., 2021). Various treatment methods have been adopted, such as relieving cough and reducing phlegm, strengthening the spleen and regulating qi, moistening dryness and resolving phlegm, nourishing yin and clearing the lungs, clearing heat, and detoxification, and are selected based on the pathway of occurrence (Koenen et al., 2023; Li et al., 2022). Clinical and related studies have shown that TCM has high cure and effective rates in treating MP, though treatment efficacy remains low in some patients (Liu et al., 2022; Ling, 2015; Liu, 2016; Wan et al., 2022). Azithromycin (AZM) is a first-line drug for the treatment of MP, although AZM shows good therapeutic effects, its efficacy is reduced over time, and some patients present gastrointestinal adverse reactions (Heidary et al., 2022). Previous studies found that the combination of TCM formulas and AZM can effectively improve the clinical symptoms in children and reduce adverse reactions (Wang et al., 2021; Zhang et al., 2021). However, existing studies have limited sample size and clinical translatability. Consequently, we performed a systematic evaluation and meta-analysis to evaluate the efficacy and safety of TCM combined with AZM in the treatment of pediatric MP, and provide an evidence-based outlook on its clinical applicability.

## Methods

The study was conducted following the Preferred Reporting Items for Systematic Reviews and Meta-Analyses (PRISMA) (Moher et al., 2015).

### Search strategy

Two reviewers independently searched seven databases including four Chinese databases such as China’s Knowledge Infrastructure (http://www.cnki.net,CNKI), the Chinese periodical service platform (http://www.cqvip.com,CQVIP), Wanfang knowledge service platform (http://www.wanfangdata.com,WF), China National Science and Technology Library and documentation center (http://www.nstl.cn,NSTL), and three English databases such as PubMed, Cochrane Library, and EMBASE.English. Following search terms: (“Traditional Chinese medicine” OR “TCM”) AND (“children” OR “pediatric”) AND (“Azithromycin”) AND (“*mycoplasma* pneumonia”) between January 1, 2000 and November 1, 2023. The detailed search strategies can be showed in Supplementary Table S1.

### Eligibility

Inclusion criteria: 1) The research topic was MP; 2) subjects were aged ≤14 years; 3) study design was a randomized controlled trial; 4) clear reporting of no significant difference between the baseline data of the experimental and control groups; 5) intervention measures were TCM decoction combined with AZM sequential therapy, and symptomatic treatment was given; 6) control measure was AZM sequential therapy combined with symptomatic treatment; and 7) reporting of at least one clear efficacy indicator or adverse reaction observation.

Exclusion criteria: 1) duplicate published literature; 2) full-text literature unavailable; 3) missing results or obvious errors in the literature.

### Literature quality assessment and data extraction

Two independent reviewers used the Cochrane risk-of-bias tool to assess the quality of the literature. Random sequence generation, allocation concealment, blinding of participants and personnel, blinding of outcome assessment, incomplete outcome data, selective reporting, and other biases were evaluated, and the risk of bias was determined as low, unclear,or high (Higgins et al., 2011). In case of disagreement, the outcome was determined after discussion with a third reviewer.

We designed a structured data extraction procedure to extract the first author, publication year, population of each group, patient characteristics, treatment measures, drug composition, and treatment results. The treatment outcomes included cure, effectiveness, fever disappearance time, cough disappearance time, pulmonary rales disappearance time, untoward reaction, and C-reactive protein (CRP), interleukin-6 (IL-6), and tumor necrosis factor-α (TNF- α) levels. “Cure” was defined as the disappearance of asthma, fever, cough, dry/wet lung rales and other post-treatment signs, and recovery in chest X-rays. “Effective” referred to a significant improvement in cough, asthma, fever, and dry/wet lung rales after treatment. Data extraction was performed by two independent reviewers, and any disagreements were resolved through discussion with the third reviewer.

### Statistical analysis

We summarized the results of the included studies and calculated the sample size, mean, and standard deviation. For dichotomous variables, such as cure, effectiveness, and untoward reaction after treatment, we calculated the odds ratio (OR) with 95% confidence interval (CI). For continuous variables, such as fever disappearance time, cough disappearance time, CRP, and IL-6 levels, we calculated the mean difference (MD) with 95% CI. Review Manager 5.3 (The Cochrane Collaboration, London, UK) was used for analysis, and I^2^ was calculated to estimate heterogeneity. Generally, an I^2^ of 25% indicates low heterogeneity, 50% indicates moderate heterogeneity, and 75% indicates high heterogeneity (Higgins et al., 2011). If the heterogeneity of multiple studies is low (I^2^ < 25%), a fixed effect model is used, and if the heterogeneity is high (I^2^ ≥ 25%), a random effects model is used. We assessed the risk of publication bias using funnel plots.

## Results

### Systematic literature search results

**FIGURE 1:**
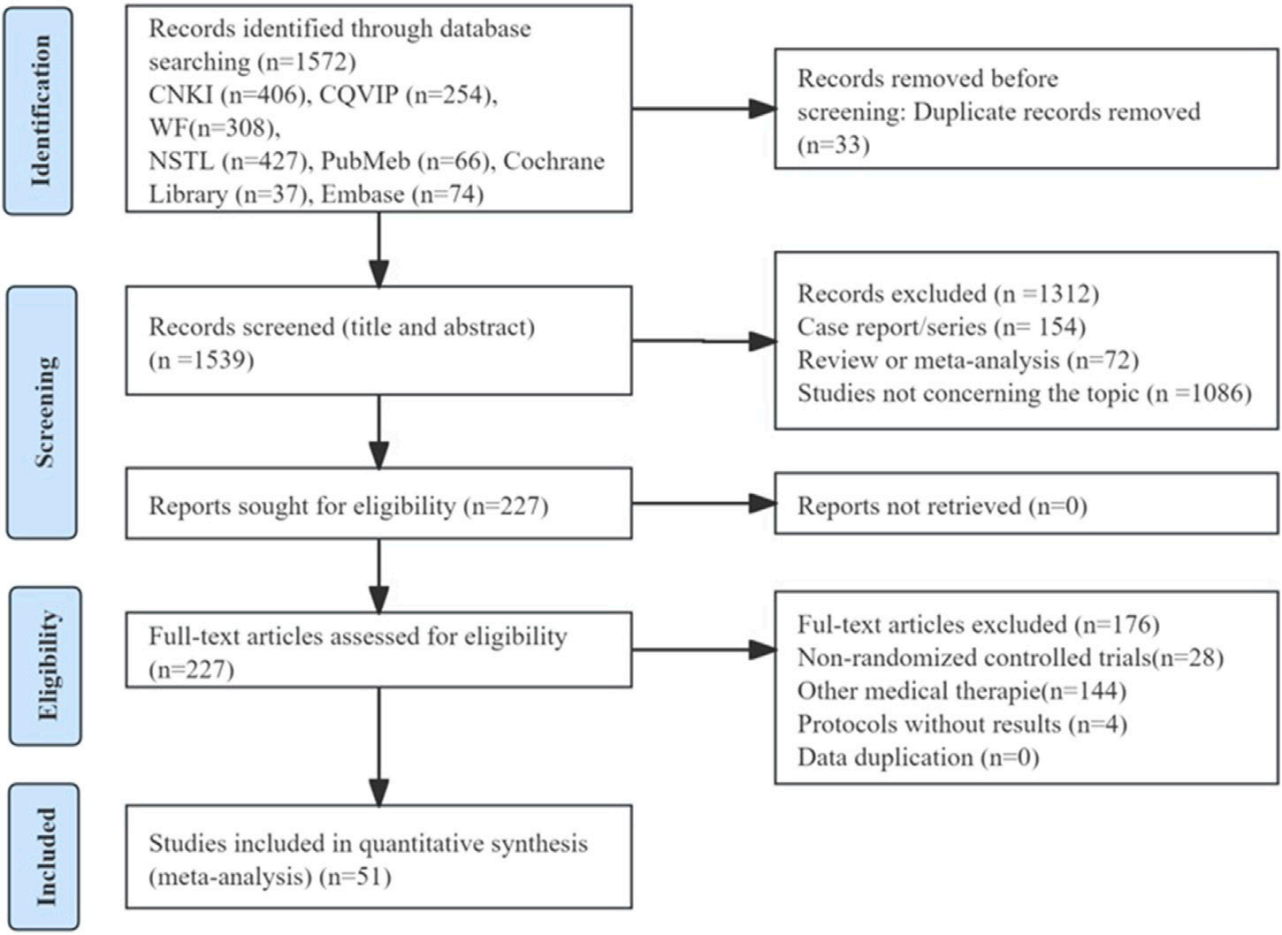
Flow diagram of study selection process.

A total of 774 potentially relevant publications were searched by electronic searching approaches. After eliminating duplicates of 1,539 records were screened. Then after excluding abstracts, the titles, case report/series, and full text of 1,539 records, finally, we included 51 RCTs with 5,799 children for systematic evaluation and meta-analysis. The screening process was shown in Figure 1. Main characteristics of the RCTs were shown in Table 1.

**TABLE 1:** Summary of studies included in the meta-analysis.

Study	Characteristics		Age		Sample size		Outcome
	Country	Year	E	C	E	C	
Cai et al. (2017)	China	2015–2016	5–13	5–13	59	59	①②⑥
Chen (2015)	China	2011–2013	1–13	1–13	60	60	①②③④⑥
Chen and Liang (2019)	China	2016–2017	3–14	3–14	43	43	②③④⑤⑥
Deng et al. (2021)	China	2018–2019	3–14	3–14	45	45	①②③④⑤⑥⑧⑨
Dong et al. (2021)	China	2014–2015	1–14	1–14	30	30	①②③④⑨
Gou et al. (2022)	China	2020–2021	3–14	3–14	51	51	①②
Han (2015)	China	2014–2015	1–14	1–14	40	40	②③④⑤⑥⑦⑧
He et al. (2017)	China	2015–2016	2–12	2–12	57	57	①②③④⑤⑥⑨
Hu et al. (2022)	China	2018–2020	1–10	1–10	40	40	③④⑤⑥
Huang et al. (2009)	China	2007–2008	2–13	2–13	40	40	①②③④⑤⑥
Li et al. (2009)	China	2005–2007	1–14	1–14	52	42	②③④⑤
Li G. et al. (2021)	China	2017–2019	1–11	1–11	50	50	①②⑦⑧⑨
Li L. et al. (2013)	China	2011–2012	1–14	1–14	41	38	①②③④⑤⑨
Li L. et al. (2021)	China	2018–2020	4–14	4–14	45	45	①②③④⑤⑥⑦⑨
Li T. (2019)	China	2016–2017	2–13	2–13	54	54	①②③④⑤
Li W. et al. (2013)	China	2009–2012	2–14	2–14	42	42	①②③④⑤
Li et al. (2015)	China	2013–2014	3–14	3–14	52	52	②③④⑤⑦⑧
Li Z. et al. (2013)	China	2011–2012	1–11	1–11	45	45	①②③④⑤⑨
Liao F. et al. (2009)	China	2006–2008	1–14	1–14	113	113	①②③④
Liao et al. (2010)	China	2009–2010	6–12	6–12	40	40	①②⑨
Liao et al. (2011)	China	2010–2011	1–12	1–12	50	50	①②③④⑤
Liu et al. (2010)	China	2006–2009	1–14	1–14	45	45	②③④⑤⑦⑧⑨
Liu (2016)	China	2015–2016	1–9	1–9	48	48	①②⑨
Liu (2016b)	China	2015–2016	2–12	2–12	41	41	①②⑥⑨
Liu et al. (2011)	China	2009–2010	1–14	1–14	52	52	①②③④⑤
Liu and Chen (2022)	China	2018–2020	5–14	5–14	101	101	①②③⑤⑦
Liu (2007)	China	2001–2006	2–12	2–12	60	60	①②③④
Lv et al. (2021)	China	2016–2019	1–10	1–10	53	53	①②③④⑤⑨
Ma et al. (2021)	China	2018–2019	3–13	3–13	34	34	①②③④⑤⑥⑦
Mao et al. (2020)	China	2019–2020	1–14	1–14	50	50	①②③④⑤⑨
Mou et al. (2018)	China	2015–2016	1–8	1–8	46	46	①②③④⑤⑥⑧⑨
Ou and Wang (2022)	China	2020–2021	1–12	1–12	103	103	②⑥⑦⑧⑨
Qi and Liu (2017)	China	2012–2014	1–14	1–14	42	42	①②③④⑤⑦⑧⑨
Qian (2011)	China	2009–2010	2–14	2–14	37	37	①②③④⑤⑦⑧
Qin (2015)	China	2011–2012	1–10	1–10	120	80	①②③⑤
Shu and Zhang (2017)	China	2013–2014	1–13	1–13	49	49	①②③④⑤⑥⑦⑧⑨
Su and Wang (2008)	China	2004–2006	1–13	1–13	40	40	①②③④⑤⑨
Sun and Dong (2022)	China	2018–2021	1–14	1–14	48	49	①②③④⑦
Wang et al. (2015)	China	2010–2013	1–9	1–9	62	62	①②③④⑤⑨
Wang and Hou (2020)	China	2018–2019	4–12	4–12	69	65	①②⑦⑧⑨
Wu and Wu (2016)	China	2013–2014	2–14	2–14	92	92	①②③④⑤⑦⑧⑨
Wu et al. (2017)	China	2015–2016	2–14	2–14	80	80	②③④⑤⑧
Xu and Shen (2019)	China	2015–2018	2–12	2–12	20	20	①②③④⑤⑥⑦⑨
Yang (2016)	China	2012–2014	1–12	1–12	50	50	①③④⑤⑥⑦⑧
Yu (2008)	China	2005–2007	1–11	1–11	58	58	①②③④⑤⑨
Zhang (2016)	China	2015–2016	1–14	1–14	65	65	①②⑥⑦⑧⑨
Zhang et al. (2018)	China	2016–2017	2–9	2–9	30	30	①②③④⑤
Zhang et al. (2017)	China	2015–2016	2–11	2–11	63	63	①②③④⑥⑨
Zhao and Zhao (2009)	China	2006–2008	0–14	0–14	263	198	①②⑨
Zuo et al. (2014)	China	2013–2013	1–13	1–13	60	60	①②③④⑤⑨

E, experimental group, traditional Chinese medicine formula combined with azithromycin treatment; C, control group, Conventional azithromycin treatment. Both groups were given symptomatic treatment. ①: Cure rate; ②: efficiency; ③: cough disappearance time; ④: heat disappearance time; ⑤: lung rale disappearance time; ⑥: CRP, level; ⑦: IL-6, level; ⑧: TNF-α, level; ⑨: adverse reactions.

### Literature quality evaluation

**FIGURE 2:**
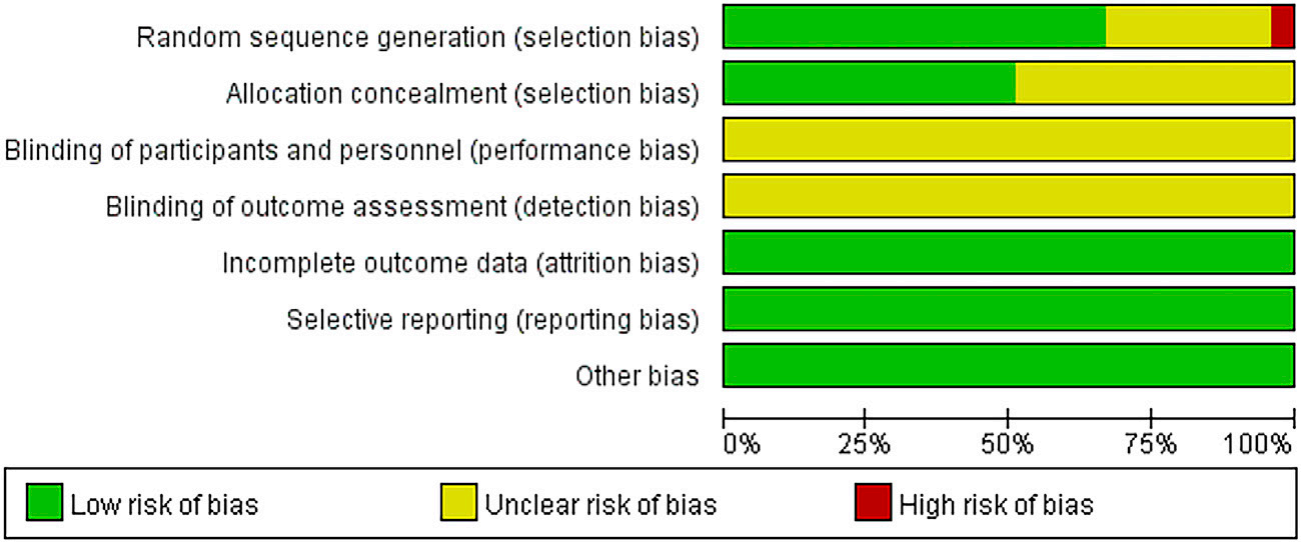
Risk of bias.

Among the 51 RCTs, 38 studies used the random number table method, 11 studies only mentioned random grouping without specifying specific methods, and 2 studies performed grouping according to the order of subject visits. None of the literature explicitly mentioned the blinding method of implementers and participants, or the blinding method used in outcome evaluation. All literature had complete outcome data, selective reporting of outcome indicators, or no other biases (Figure 2).

### Treatment effect

**FIGURE 3:**
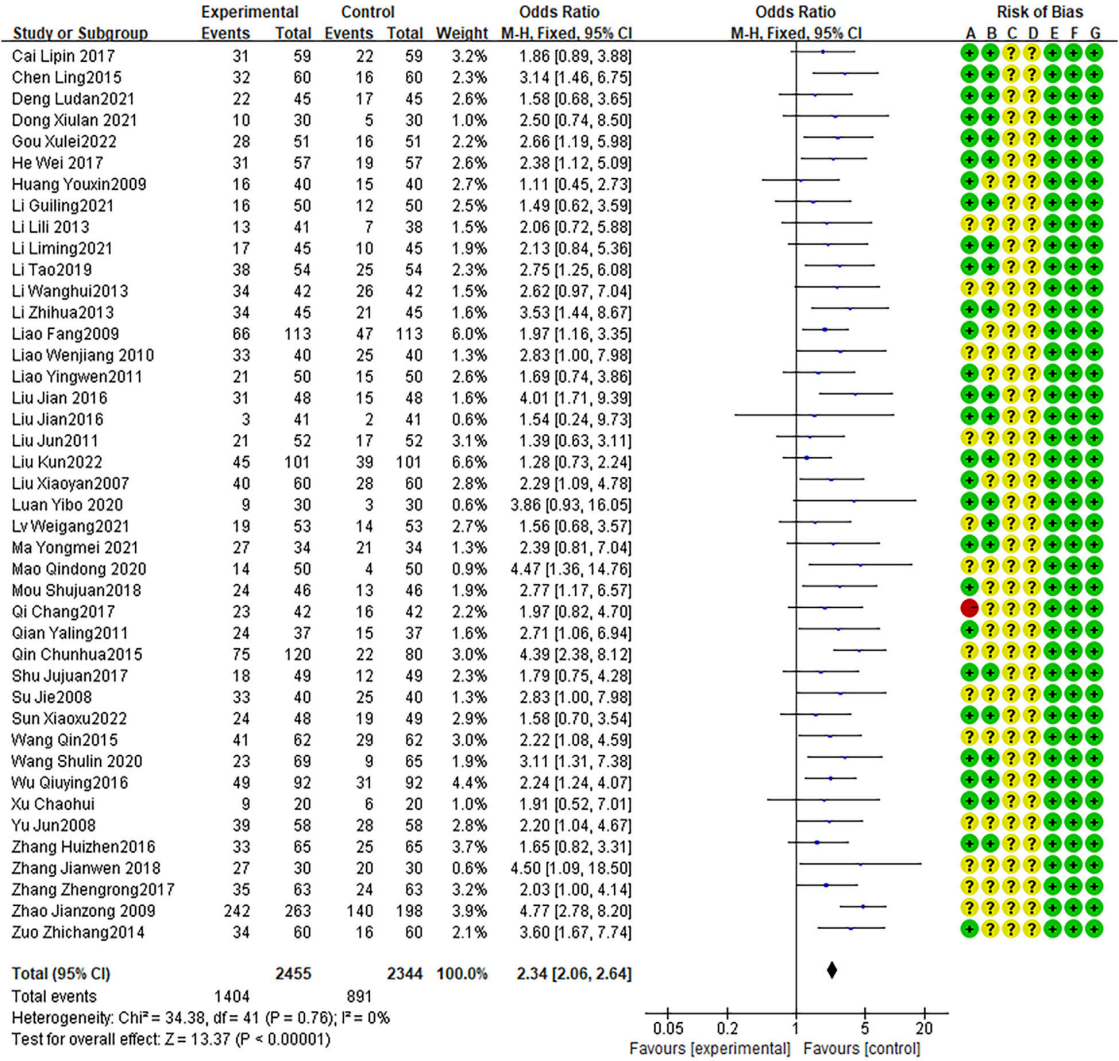
Meta-analysis result of cure rate.

**FIGURE 4:**
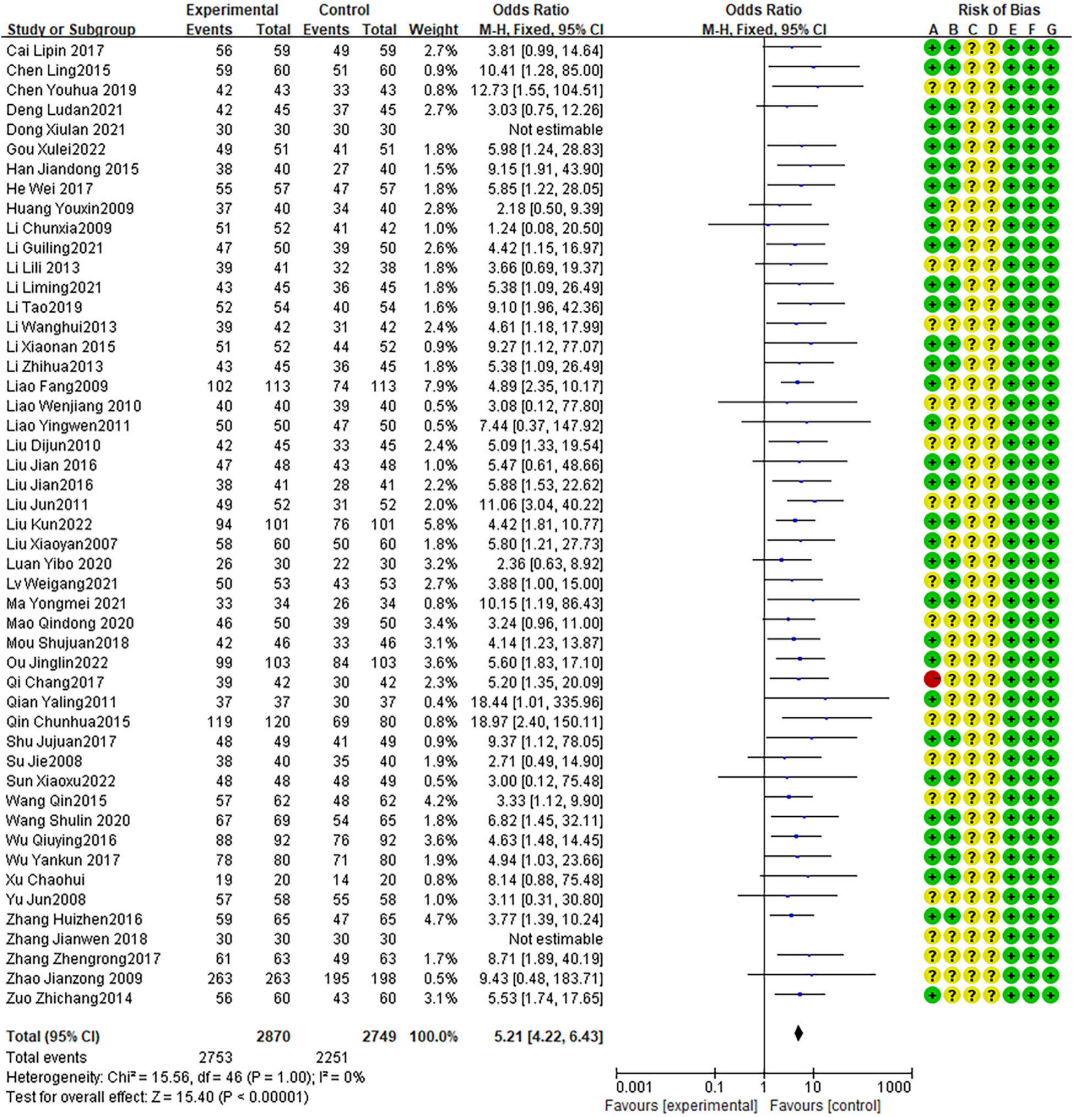
Meta-analysis result of effective rate.

Forty-two articles reported cure rates, with no heterogeneity between studies (I2 = 0%, P = 0.76). The fixed effects model indicated that the therapeutic effect of the combination therapy was better than that of AZM alone (OR = 2.34, 95% CI: 2.06 to 2.64, P < 0.0001; Figure 3). In addition, 49 RCTs reporting effective rates, with no heterogeneity between studies (I2 = 0%, P = 1.00). The fixed effects model indicated that the effective rate of the combination therapy was higher than that of the AZM treatment (OR = 5.21, 95% CI: 4.22 to 6.43, P < 0.0001; Figure 4).

### Cough disappearance time

**FIGURE 5:**
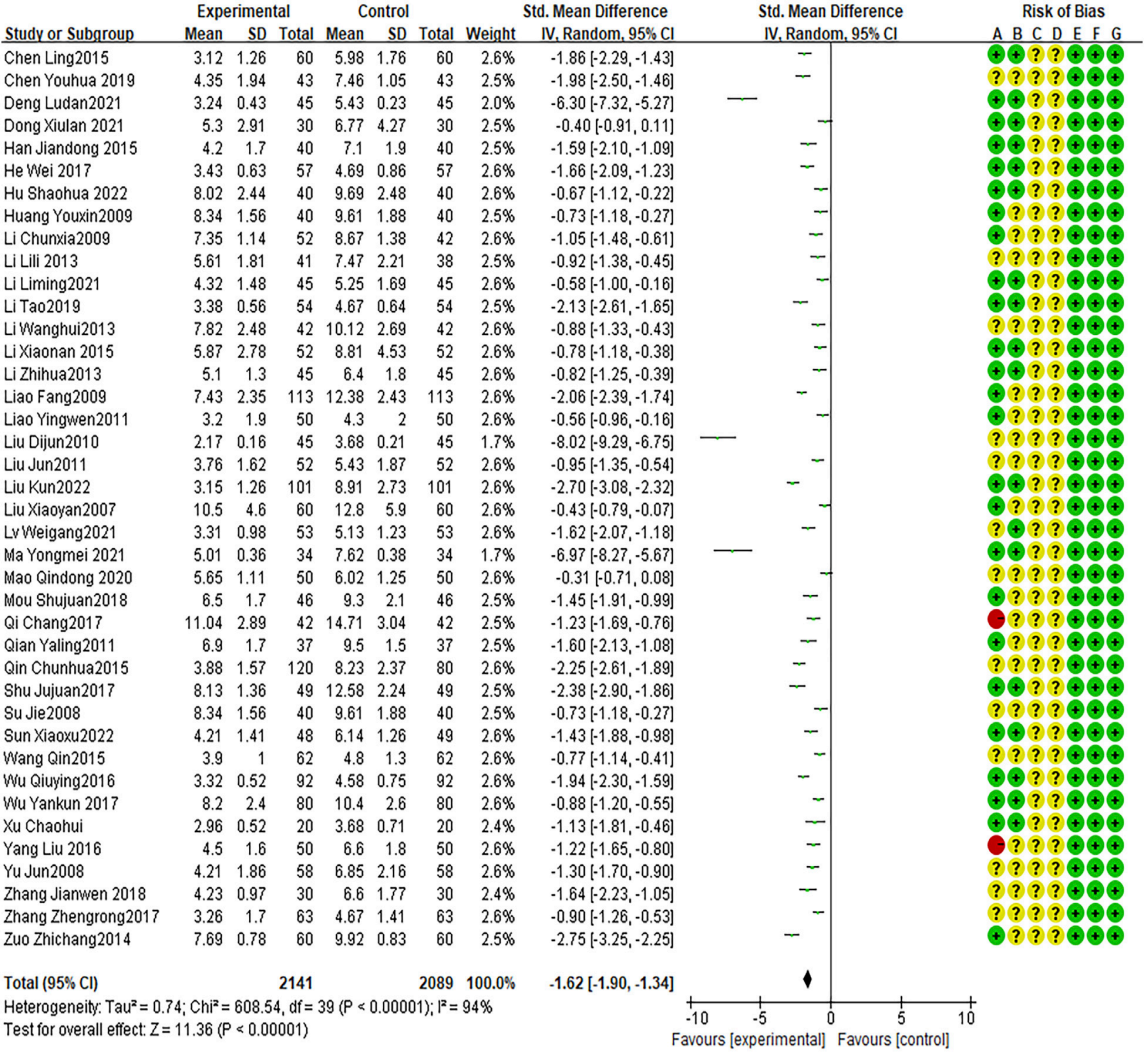
Meta-analysis result of cough disappearance time.

Forty RCTs reported cough disappearance time, with high heterogeneity between studies (I2 = 94%, P < 0.0001). The random effects model indicated that the average cough disappearance time was shorter with combination therapy than that with AZM therapy (WMD = −1.62, 95% CI: −1.90 to −1.34, P < 0.0001; Figure 5).

### Duration of fever

**FIGURE 6:**
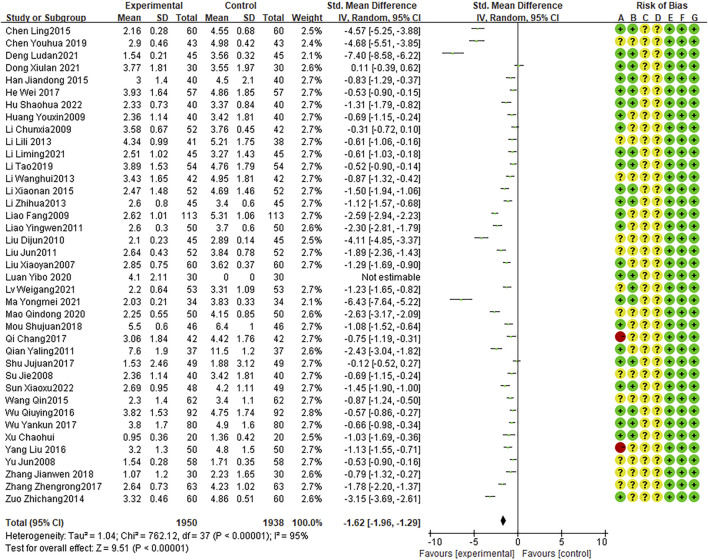
Meta-analysis result of duration of fever.

Twenty-four RCTs presented the duration of fever, with high heterogeneity between studies (I2 = 95%, P < 0.0001). The random effects model indicated that the average duration of fever was shorter with combination therapy than that with AZM therapy (WMD = −1.62, 95% CI: −1.96 to −1.29, P < 0.0001; Figure 6).

### Disappearance time of lung rales

**FIGURE 7:**
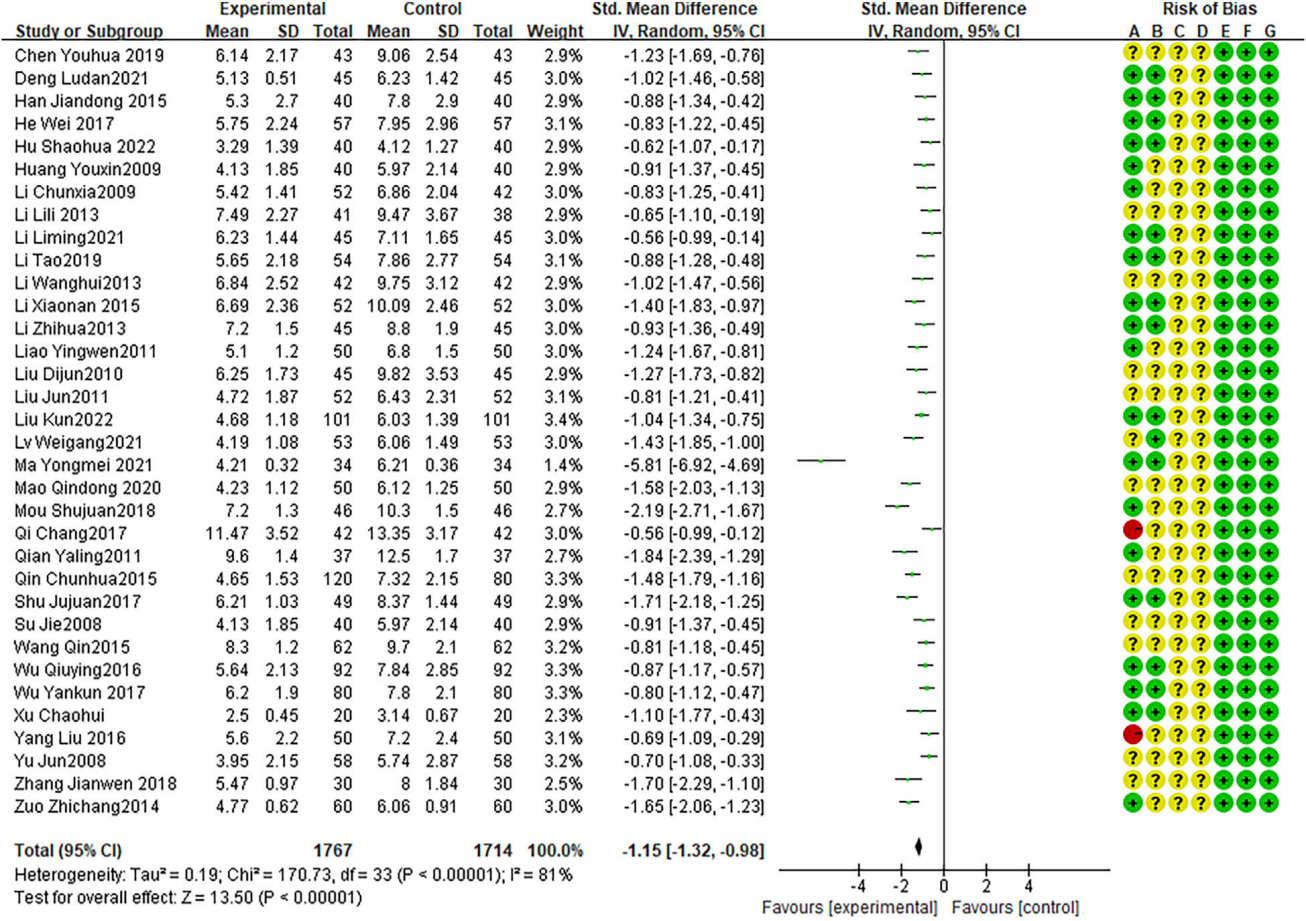
Meta-analysis result of disappearance time of lung rales.

Twenty-two RCTs presented the disappearance time of lung rales, with high heterogeneity among RCTs (I2 = 81%, P < 0.0001). The random effects model indicated that the average disappearance time of lung rales was shorter with combination therapy than that with AZM therapy (WMD = −1.15, 95% CI: −1.32 to −0.98, P < 0.0001; Figure 7).

### Post-treatment CRP levels

**FIGURE 8:**
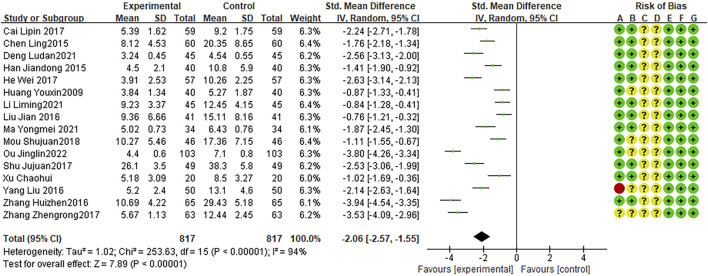
Meta-analysis result of post-treatment CRP Levels.

Sixteen RCTs reported CRP levels after treatment, with high heterogeneity among studies (I2 = 94%, P < 0.0001). The random effects model indicated that the average CRP level after treatment was lower after combination therapy than that after AZM therapy (WMD = −2.06, 95% CI: −2.57 to −1.55, P < 0.0001; Figure 8).

### Post-treatment IL-6 levels

**FIGURE 9:**
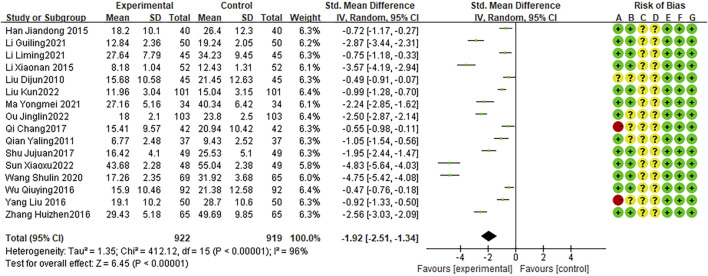
Meta-analysis result of post-Treatment IL-6 Levels.

Sixteen RCTs reported IL-6 levels after treatment, with high heterogeneity among studies (I2 = 96%, P < 0.0001). The random effects model indicated that the average IL-6 level in was lower after combination treatment than that after AZM treatment (WMD = −1.92,95% CI: −2.51 to −1.34, P < 0.0001; Figure 9).

### Post-treatment TNF-α levels

**FIGURE 10:**
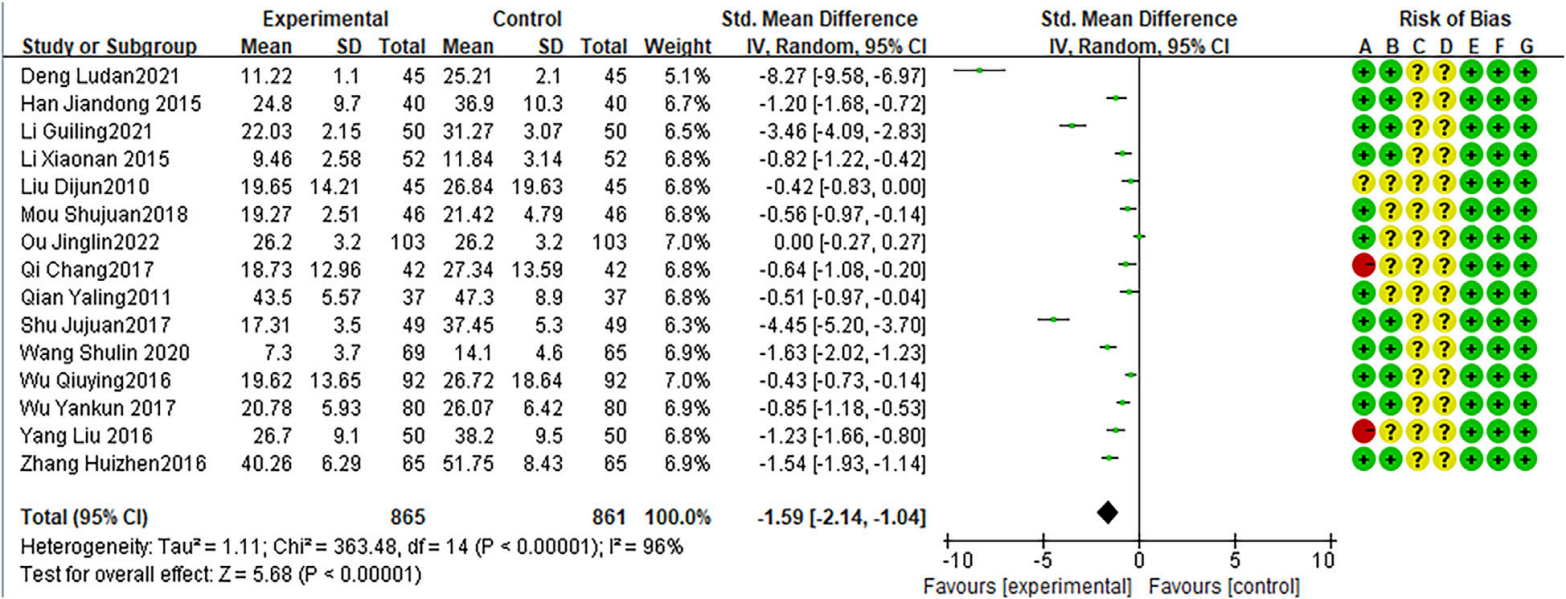
Meta-analysis result of post-treatment TNF-α levels.

Fifteen RCTs reported post-treatment TNF-α levels, with high heterogeneity between studies (I2 = 96%, P < 0.0001). The random effects model indicated that the average TNF-α level was lower after combination therapy than that after AZM therapy (WMD = −1.59, 95% CI: −2.14 to −1.04 P < 0.0001; Figure 10).

### Adverse reactions

**FIGURE 11:**
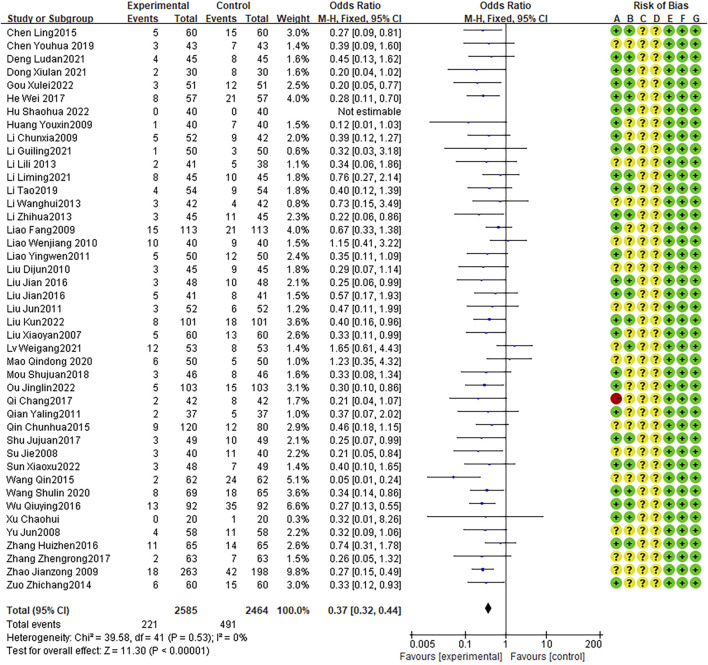
Meta-analysis result of adverse reactions.

Forty-three RCTs demonstrated adverse reactions, with no heterogeneity between studies (I2 = 0%, P = 0.53). The fixed effects model indicated that the number of adverse reactions was lower with combination therapy than that with AZM therapy (OR = 0.37, 95% CI: 0.32 to 0.44, P < 0.0001; Figure 11).

### Publication bias

**FIGURE 12:**
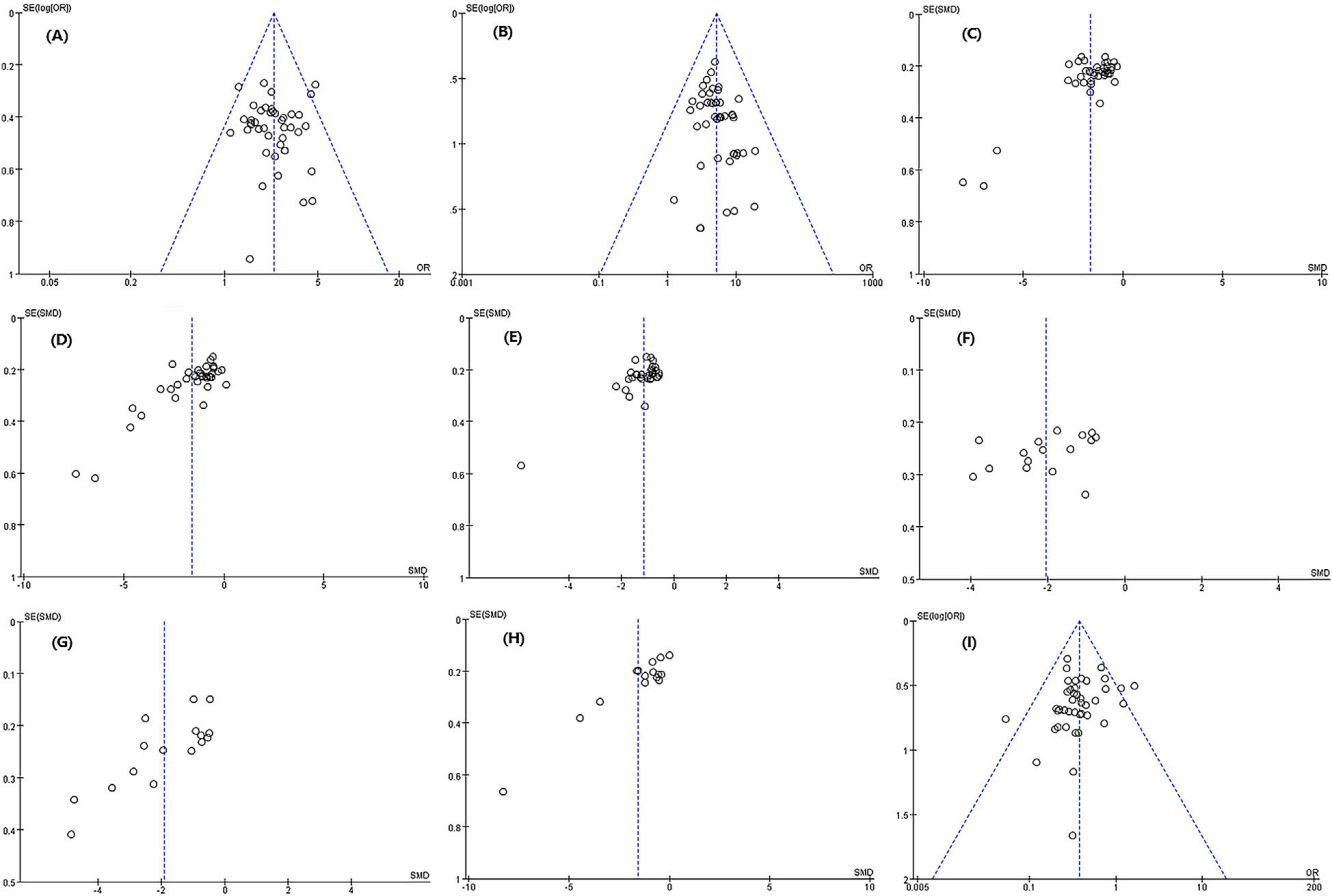
Meta-analysis result of publication bias.

The publication bias between cure rate, effective rate, disappearance time of lung gong sounds, post-treatment CRP level, and incidence of adverse reactions was relatively small. There was a certain degree of publication bias in cough disappearance time, fever disappearance time, IL-6 level after treatment, and TNF-α level after treatment (Figure 12).

### Usage of Chinese Medicinal materials

Among the 51 RCTs, a total of 115 Chinese medicinal herbs were used in the combination therapy. The top ten Chinese medicinal herbs with the highest frequency of use were Licorice, almond, *Scutellaria baicalensis*, Ephedra, Fritillaria, plaster stone, mulberry root bark, *Platycodon grandiflorus*, Lepidium seed, *Houttuynia cordata*, and Gualou. The statistical results of the main Chinese medicinal materials and their usage frequency are shown in Table 2.

**TABLE 2:** Main Chinese medicinal materials and frequency of use.

Name	Frequency	Name	Frequency	Name	Frequency
Licorice	48	Raphanus seed	10	Tangerine peel	5
Almond	44	Poria cocos	9	Figwort root	5
Scutellaria baicalensis	36	Honeysuckle	9	Atractylodes macrocephala	4
Ephedra	35	Peach kemel	9	Eriobotryae folium	4
Fritillary	28	Tatarian aster root	9	Peucedani radix	4
Plaster Stone	25	Forsythia suspensa VAHL	8	Folium mori	4
Mulberry root bark	15	Cicada Slough	7	Rehmanniae radix	4
Platycodon grandiflorum	14	Adenophora stricta	7	Radices paeoniae alba	3
Lepidium Seed	14	Anemarrhena asphodeloides	7	Radix bupleuri	3
Houttuynia cordata	14	Fructus aurantii	7	Radix et rhizoma	3
Fructus trichosanthis	14	Salvia	6	Semen benincasae	3
Radix stemonae	11	Pheretima	6	Bombyx batryticatus	3
Pinellia ternata	11	Giant knotweed rhizome	6	Herba schizonepeta	3
Dwarf lilyturf tuber	11	Fructus gardeniae	6	Reed rhizome	3
Perilla seed	11	Bamboo shavings	6	Moutan cortex	3

## Discussion

In this study, we conducted a meta-analysis and evaluation of 51 RCTs with 5,799 MP among children, the findings showed that TCM combined with AZM in fighting to pediatric MP had favorable efficacy and safety. We also found that the combination treatment can improve the cure and effective rates, shorten the disappearance time of cough, fever, and lung rales, and reduce CRP, IL-6, and TNF-α levels, representing an overall positive treatment effect in children with MP.

Previous meta-analyses on the treatment of pediatric MP with TCM combined with AZM mostly focused on the efficacy and safety of traditional Chinese patent medicines and simple preparations (He et al., 2020; Sun et al., 2020; Wei et al., 2020). To our knowledge, this is the first meta-analysis to evaluate the efficacy of various TCM combined with AZM in the treatment of pediatric MP. Although traditional Chinese patent medicines, simple preparations, and TCM are prescribed according to the etiology, pathogenesis, and clinical symptoms, there are some differences between traditional Chinese patent medicines/simple preparations and TCM. In traditional Chinese patent medicines and simple preparations, the ingredients are basically fixed, while the ingredients in TCM are adjusted according to patient symptoms for personalized targeted treatment (Zhang et al., 2021). Although the meta-analysis of traditional Chinese patent medicines and simple preparations combined with AZM in the treatment of pediatric MP can aid in clinical decision-making, it does not identify the most effective.

Chinese herbal formulas. Changes in the composition of TCM pose potential risks (Salmerón et al., 2021). In the 51 RCTs, some TCM ingredients were identified with potential safety effects, such as almonds, *Scutellaria baicalensis*, ephedra, *Pinellia ternata*, Tianlizi, and rhubarb, among others. Although these drugs may promote adverse reactions (Sun et al., 2020), we found that the combination of TCM and AZM did not correspond with an increase in adverse reactions—the incidence of adverse reactions was reduced compared with using AZM alone, and there were no cases of liver dysfunction. The reason is that all TCM ingredients are processed and produced, and the amount prescribed in treatment is relatively small. Therefore, adverse reactions are unlikely without excessive intake (Doan et al., 2020).

The main types of Chinese medicinal materials in the 51 RCTs were prescribed for lung clearing, phlegm resolving, cough relieving, heat clearing, and antiviral effects. These medicinal herbs contain active ingredients that can inhibit the production of the fever mediators PGE2 and GAMP, promote Th2 cell differentiation, increase anti-inflammatory factor levels, and regulate immune function, thereby enhancing physical function (Hart et al., 2022). The TCMs might play a role in accelerating symptom relief, promoting disease recovery, and reducing the gastrointestinal discomfort caused by AZM.

Our study has several limitations. Firstly, the selection of subjects was based on children admitted to hospitals where the investigators worked. The lack of multicenter research may promote bias in the selection of subjects. Secondly, some indicators had high heterogeneity such as CRP, IL-6, and TNF-a, which may be related to the inconsistent baseline data of each RCT, such as patient age, disease duration, and severity. In addition, the differences in TCM components and biochemical detection techniques used in different RCTs might also lead to high heterogeneity of the above indicators.

## Conclusions

The combination of TCM and AZM in the treatment of pediatric MP could significantly improve the cure and effective rates, promote symptom relief, reduce the concentration of inflammatory factors, and reduce the occurrence of adverse reactions. It may be the best treatment choice for pediatric MP. More high-quality multicenter researches should need to be conducted in the future, and further confirm the findings.

## Data Availability

The original contributions presented in the study are included in the article/Supplementary Material, further inquiries can be directed to the corresponding author.
