# Supplementary material for: Efficacy and safety of traditional Chinese medicine combined with azithromycin sequential therapy for mycoplasma pneumonia among children: a meta-analysis of randomized controlled trials
Source: Front Pharmacol. 2024 Oct 17;15:1431706. doi: 10.3389/fphar.2024.1431706 (PMC11524904; doi:10.3389/fphar.2024.1431706)
Supplement: Supplementary file 1 [file Table1.docx]

**Supplementary Table**

**Table S1. Search strategies in each database**

| **Database** | **Search terms** | **Number** |
| --- | --- | --- |
| PubMeb | ((Traditional Chinese medicine [Title/Abstract] OR TCM [Title/Abstract]) AND (children [Title/Abstract] OR pediatric [Title/Abstract]) AND (azithromycin [Title/Abstract]) AND (mycoplasma pneumonia [Title/Abstract])) | 66 |
| CochraneLibrary | ((Traditional Chinese medicine [Title/Abstract] OR TCM [Title/Abstract]) AND (children [Title/Abstract] OR pediatric [Title/Abstract]) AND (azithromycin [Title/Abstract]) AND (mycoplasma pneumonia [Title/Abstract])) | 37 |
| Embase | ((Traditional Chinese medicine [Title/Abstract] OR TCM [Title/Abstract]) AND (children [Title/Abstract] OR pediatric [Title/Abstract]) AND (azithromycin [Title/Abstract]) AND (mycoplasma pneumonia [Title/Abstract])) | 74 |
| CNKI | ((题名或关键词="支原体肺炎")AND(题名或关键词="儿童") AND (题名或关键词"中医药”) AND (题名或关键词"阿奇霉素")) | 406 |
| CQVIP | ((题名或关键词="支原体肺炎")AND(题名或关键词="儿童") AND (题名或关键词"中医药”) AND (题名或关键词"阿奇霉素")) | 254 |
| WANFANG | ((题名或关键词="支原体肺炎")AND(题名或关键词="儿童") AND (题名或关键词"中医药”) AND (题名或关键词"阿奇霉素")) | 308 |
| NSTL | ((题名或关键词="支原体肺炎")AND(题名或关键词="儿童") AND (题名或关键词"中医药”) AND (题名或关键词"阿奇霉素")) | 427 |

* Chinese periodical service platform, CQVIP; China's Knowledge Infrastructure, CNKI; WanFang, China National Science and Technology Library (NSTL); China National Science and Technology Library, NSTL.
